# Peripapillary Vessel Density Mediates the Relationship Between Axial Length and Visual Field Damage in Glaucoma

**DOI:** 10.3390/jcm15093555

**Published:** 2026-05-06

**Authors:** Samuel Potash, Alon Harris, Alice Verticchio Vercellin, Keren Wood, Gal Yaakov Cohen, Adam Rosen, Minwoo Kwon, Jacob Rothstein, Yash Lahoti, Lucia Tanga, Carmela Carnevale, Brent A. Siesky

**Affiliations:** 1 Department of Ophthalmology, Icahn School of Medicine at Mount Sinai, New York, NY 10029, USA; samuel.potash@mssm.edu (S.P.); alice.verticchio@mssm.edu (A.V.V.); keren.woodshalem@mssm.edu (K.W.); gal.cohen@mountsinai.org (G.Y.C.); adam.rosen@mountsinai.org (A.R.); minwoo.kwon@icahn.mssm.edu (M.K.); jacob.rothstein@mssm.edu (J.R.); yash.lahoti@icahn.mssm.edu (Y.L.); 2 Goldschleger Eye Institute, Tel Hashomer 52621, Israel; 3 Sheba Medical Center, Ramat Gan 52621, Israel; 4 Tel Aviv University, Tel Aviv 69978, Israel; 5 SUNY Downstate Health Sciences University, Brooklyn, NY 11203, USA; 6 Yeshiva University, New York, NY 10003, USA; 7 IRCCS-Fondazione Bietti, 00184 Rome, Italy; lucia.tanga@fondazionebietti.it (L.T.); carmela.carnevale@fondazionebietti.it (C.C.)

**Keywords:** glaucoma, imaging, optic nerve, myopia

## Abstract

**Objectives:** Axial myopia is a risk factor for glaucoma, yet the mechanism(s) linking axial length (AL) to visual field (VF) damage remain unclear. This pilot study investigates to what extent peripapillary vessel density (pVD) mediates the relationship between AL and VF outcomes in patients with primary open-angle glaucoma (POAG). **Methods:** This cross-sectional pilot study included a primary cohort of 26 POAG eyes with directly measured AL, with an exploratory expanded cohort of 71 POAG eyes used to assess the consistency of findings. Optical coherence tomography angiography quantified pVD across eight sectors. VF outcomes included mean deviation, pattern standard deviation, and visual field index. Mediation analyses used ordinary least squares regression adjusted for age, intraocular pressure, and scan quality. Indirect effects were assessed via bias-corrected bootstrap confidence intervals. **Results:** In both primary and expanded cohorts, inferior-temporal pVD mediated the relationship between AL and all VF outcomes. Additional sectors emerged as mediators in the expanded cohort, suggesting broader vascular involvement. **Conclusions:** Peripapillary microvascular compromise significantly mediates the impact of AL on glaucomatous VF loss, supporting a possible biomechanical–vascular model of POAG eyes across a range of axial lengths.

## 1. Introduction

Glaucoma is a heterogeneous group of progressive optic neuropathies characterized by retinal ganglion cell (RGC) loss, excavation of the optic nerve head (ONH) and corresponding visual field (VF) defects. The disease is the leading cause of irreversible blindness worldwide, currently affecting more than 70 million people [1]. Established risk factors for glaucoma include advancing age, family history, African and Latino ancestry, thin central corneal thickness (CCT), elevated intraocular pressure (IOP) and structural features such as large cup-to-disc ratio and increased axial length (AL) [2]. Although elevated IOP remains the only approved modifiable risk factor, glaucoma can occur across a broad IOP spectrum, reflecting its multifactorial pathophysiology. Normal-tension glaucoma (NTG), a subtype of primary open-angle glaucoma (POAG), demonstrates characteristic optic neuropathy and VF loss despite statistically normal/low IOP. The presence of NTG underscores the role of non-IOP factors in glaucoma pathogenesis, including vascular dysfunction [3], impaired autoregulation [4], and microvascular compromise [5].

Myopia is an increasingly common refractive condition in which the eye focuses light in front of the retina, primarily driven by excessive axial elongation. Numerous epidemiologic and clinical studies demonstrate that myopia is associated with an increased risk of glaucoma, as well as more challenging diagnosis and monitoring [6,7]. Structural remodeling in myopic eyes, including scleral thinning, peripapillary atrophy, and tilted, shallowly inserted optic nerves, can mimic, obscure, and potentiate glaucomatous features [8]. Axial elongation also stretches and deforms the ONH and lamina cribrosa, increasing biomechanical strain that correlates with faster RNFL thinning and more rapid glaucoma progression [5,6,7,8,9,10,11]. Beyond mechanical vulnerability, vascular mechanisms are also implicated in linking AL to glaucoma, with longer eyes showing greater reductions in retinal microcirculation [12], choroidal blood flow [13], macular and peripapillary capillary density [14], and peripapillary vasoreactivity [15].

Optical coherence tomography angiography (OCTA) provides noninvasive, high-resolution visualization of retinal and ONH microvasculature. Recent OCTA studies have provided growing evidence of vascular involvement in both glaucoma and myopia. Specifically in glaucoma, peripapillary vessel density (pVD) is reduced compared with healthy controls, and these reductions correlate strongly with structural measures such as RNFL thickness and functional VF indices [16]. In myopic eyes, OCTA studies demonstrate lower pVD even in the absence of glaucoma, suggesting that axial elongation alone impairs vascular supply [17]. However, the extent to which microvascular changes help mediate the relationship between AL and functional damage in glaucoma remains poorly understood. We hypothesize that axial elongation contributes to glaucomatous progression indirectly, by reducing pVD and thereby impairing ONH perfusion. pVD was selected as the primary mediator given its well-established role as a glaucoma biomarker across defined anatomical sectors corresponding to the arcuate nerve fiber bundles most vulnerable to glaucomatous injury, and its demonstrated sensitivity to axial elongation even in non-glaucomatous eyes.

In this study, we investigated the significance of pVD in the mediation of the association between AL and glaucomatous VF damage. Unlike prior studies that have described pairwise associations between AL, pVD, and VF damage, this study is the first to formally quantify the indirect pathway from AL to VF damage via pVD using mediation analysis. Specifically, we examined the global mean deviation (MD), localized pattern standard deviation (PSD), and visual field index (VFI) as outcomes to better understand the interplay between biomechanical elongation and microvascular compromise in the pathophysiology of POAG.

## 2. Materials and Methods

This cross-sectional observational study included 150 POAG patient eyes and 150 non-glaucomatous controls and was conducted at the Department of Ophthalmology, Icahn School of Medicine at Mount Sinai, New York, NY, USA. The study was approved by the Institutional Review Board of the Icahn School of Medicine at Mount Sinai (STUDY-20-00198, approval date 19 April 2020) and adhered to the tenets of the Declaration of Helsinki. Written informed consent was obtained from all participants.

This analysis employed three distinct cohorts: (1) a primary mediation cohort of 26 POAG eyes with directly measured AL, which forms the basis of the main findings; (2) an AL estimation cohort of 27 eyes used solely to develop a model for estimating AL from spherical equivalent (SE) and age; and (3) an exploratory expanded cohort of 71 POAG eyes incorporating both measured and estimated AL, used to assess consistency of findings from the primary cohort.

### 2.1. Inclusion and Exclusion Criteria

All participants were 21 years old or older. POAG status was confirmed by a certified ophthalmologist (glaucoma specialist) regardless of the level of IOP based on gonioscopic presence of an open angle and characteristic structural and functional damage. The non-glaucomatous control subjects included participants who were free of any eye disease. The exclusion criteria for both POAG and non-glaucomatous controls were: refractive error > +9 Diopters and < −9 Diopters in SE; evidence of exfoliation or pigment dispersion; eye disease other than glaucoma; use of ocular medications (other than IOP lowering medications for glaucomatous patients or eye lubricants for dry eye); neurological disease; psychosis or other diseases that could prevent reliable eye exams; and severe, as well as unstable or uncontrolled cardiovascular, renal, or pulmonary disease. The non-glaucomatous control subjects (*n* = 150) were recruited as part of a broader study to establish normal vessel density (VD) reference ranges but were not included in the mediation analyses presented here. Mediation models test whether pVD mediates the relationship between AL and glaucomatous VF damage, which is absent in controls (making the outcome variable undefined). A small subset of controls (*n* = 4) contributed to the AL estimation model development.

### 2.2. Clinical Examinations and Testing

All study subjects underwent a single 2 h study visit including: history/questionnaire, visual acuity testing, slit-lamp biomicroscopy, heart rate, blood pressure (BP) measurements (including systolic and diastolic BP and calculation of the mean arterial pressure (MAP), via automated ambulatory cuff after 5 min rest); IOP assessment (via Goldmann applanation tonometry), VF and OCTA testing.

### 2.3. Visual Field Testing

Standard automated perimetry was performed using the Humphrey Field Analyzer through the Swedish Interactive Threshold Algorithm 24–2 test with the Humphrey VF analyzer 750i; Carl Zeiss Meditec, Inc., Oberkochen, Germany, MD, PSD, and VFI were extracted. Tests with fixation losses ≥33% or false-positive/false-negative errors >20% were excluded.

### 2.4. Optical Coherence Tomography Angiography

pVD was measured using RTVue XR Avanti SD-OCT device with AngioVue software (RTVue XR, Version 2018.1.1.63, Optovue Inc., Fremont, CA, USA). pVD was quantified in the superficial radial peripapillary capillary (RPC) slab using the automated AngioDisc protocol; as vessel segmentation is fully algorithmic with no manual grading step, conventional intra- and inter-rater variability does not apply. VD represents the percentage of area occupied by blood vessels in this slab. Measurements were acquired from 8 sectors of the peripapillary region (defined by 2 rings of 2 mm and 4 mm centered on optic disc center): superior-nasal (SN), nasal-superior (NS), nasal-inferior (NI), inferior-nasal (IN), inferior-temporal (IT), temporal-inferior (TI), temporal-superior (TS), and superior-temporal (ST). Scans with automated scan quality scores ≥4 were included to maximize sample size in this pilot study. Scan quality was included as a covariate in all mediation models to account for quality-related variance, and additional sensitivity analysis examined whether findings were robust to manufacturer-recommended quality standards (scan quality ≥ 6).

### 2.5. Retrospective Data

AL from ocular biometry, CCT, and SE (calculated as the sphere plus half the cylinder) were collected retrospectively from electronic medical records. When available, AL was measured using either the Lenstar LS 900 (Haag-Streit AG, Köniz, Switzerland) or the IOLMaster 700 (Carl Zeiss AG, Oberkochen, Germany). CCT was obtained using ultrasound pachymetry. SE was derived from automated refraction or manifest refraction documented in the clinical record.

### 2.6. Patient Cohorts

In this analysis, three cohorts of study subjects were considered. Cohort one (primary mediation cohort) comprised 26 POAG eyes (from 26 subjects, one eye per patient) (after excluding 1 eye with scan quality < 4) with clinical and imaging data, including IOP, CCT, MAP, VF indices (MD, PSD, VFI), AL, and OCTA pVD measurements in 8 sectors. Cohort 2 (ordinary least squares (OLS) AL estimation cohort) comprised 27 eyes from 27 subjects (23 with POAG, 4 non-glaucomatous controls) with both measured AL and SE and was used to develop an AL estimation model. This cohort was mixed by design to improve the range of SE and AL values for model development; it was not used in mediation analyses. Cohort three (expanded cohort) comprised 71 glaucomatous eyes (from 71 subjects, one eye per patient) (after excluding 5 eyes with scan quality < 4) which included the original 26 subjects from cohort one, plus 45 additional glaucomatous eyes with estimated AL derived from the AL estimation model. Cohort 3 had matching clinical/imaging variables as cohort one and provides preliminary support. Given that AL was estimated rather than measured for 63% of eyes in this cohort, findings from this analysis should be considered exploratory.

### 2.7. Statistical Analysis

**Figure 1:**
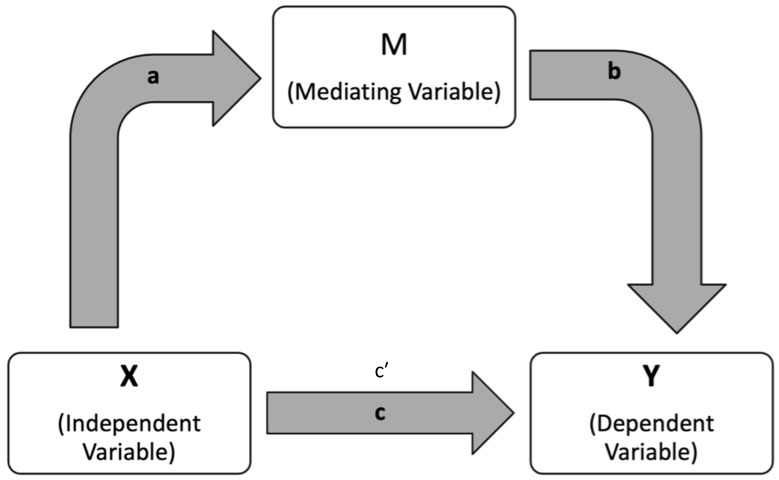
Mediation analysis pathway diagram. Paths a, b, c, and c′ represent regression coefficients quantifying the total (c), indirect (a × b), and direct (c′) effects of the predictor on the outcome through the mediator. Arrows indicate the direction of tested associations.

Mediation analyses were conducted using OLS regression in Python (v3.12.13; Google Colaboratory, Google LLC, Mountain View, CA, USA). For each VF outcome (MD, PSD, and VFI), the indirect effect of AL via pVD was calculated as the product of path coefficients (a × b). Bias-corrected bootstrap confidence intervals (5000 resamples) were used to assess significance. Models were adjusted for age, IOP, and scan quality. Multicollinearity was assessed using variance inflation factors (VIF), with values < 5 considered acceptable. For each mediator, the following paths were estimated and can be visualized in Figure 1:

- Path a: AL → pVD.
- Path b: pVD → VF outcome (MD, PSD, or VFI), controlling for AL.
- Path c: AL → VF outcome (total effect).
- Path c′: AL → VF outcome, controlling for pVD (direct effect).
- Indirect effect: Calculated as the product of coefficients (a × b).

### 2.8. Exploratory Expanded Analysis

Because AL data was not available for all participants, we estimated it using an OLS model based on SE and age: Estimated AL = 20.3629 + (−0.3545 × SE) + (0.0547 × Age). This model was applied to generate a larger sample (*n* = 71). Mediation analyses were repeated in this expanded dataset (*n* = 71 after quality exclusions) to assess whether mediation patterns observed in the primary cohort remained consistent. Results from the expanded cohort should be interpreted as hypothesis-generating given the measurement error inherent in estimated AL values.

### 2.9. Sensitivity Analyses

(1) Mediation analyses were repeated after excluding scans with scan quality <6 to assess the robustness of findings to image quality. (2) To address potential confounding by ocular magnification, we performed a sensitivity analysis using magnification-resistant relative pVD mediators, defined as each sector’s absolute value minus the mean of the other seven sectors. This approach removes global (all-sector) shifts attributable to AL-dependent magnification effects, isolating sector-specific vascular signals.

## 3. Results

The demographic characteristics (age, sex, race), eye laterality, IOP, CCT, MAP, SE, AL, VF indices (VFI, MD, PSD), and OCTA pVD of the primary and expanded cohorts are summarized in Table 1. There were no statistically significant differences between the two cohorts in any of the examined characteristics (all *p* > 0.05). The primary cohort (*n* = 26) had a mean age of 72.42 ± 7.41 years and was 50% male, while the expanded cohort (*n* = 71) had a mean age of 69.10 ± 8.88 years and was 40.8% male. Eye laterality and mean IOP were similar across cohorts, as was mean AL, with the expanded cohort utilizing combined measured/estimated AL.

The OLS AL estimation set used for the AL estimation of the expanded cohort is summarized in Table 2, including age, sex, race, laterality, AL, and SE. Our OLS regression model of AL on SE, controlling for age, demonstrated good predictive accuracy (R^2^ = 0.6263, F = 20.11, *p* = 7.42 × 10^−6^), with both SE (*p* = 2.14 × 10^−6^) and age (*p* = 0.0046) as significant predictors. However, the 95% prediction interval of ±1.49 mm indicates substantial measurement uncertainty.

Mediation analyses examining the influence of pVD across eight sectors on the relationship between AL and VF outcomes are summarized in Table 3. The table presents coefficients for paths a, b, c, and c′, along with indirect effects and their 95% confidence intervals. Significant mediation was observed for all three VF metrics (MD, PSD, and VFI) primarily through IT pVD, which consistently demonstrated the strongest indirect effects. Additionally, NI pVD demonstrated significant mediation of MD. VIF analysis revealed no concerning multicollinearity. VIF values were generally <2, with the highest observed for age (2.6) and IN pVD (3.1), both well below conventional thresholds of concern.

Identical mediation analyses performed on the expanded cohort are reported in Table 4. These results support the IT pVD as a consistent mediator across all VF indices and NI pVD as a mediator for MD. Additionally, the ST pVD emerged as a significant mediator for all VF indices.

To assess whether mediation findings were influenced by scan quality, we repeated analyses using a stricter quality threshold (scan quality ≥ 6), which is commonly recommended in the OCTA literature. After excluding lower-quality scans, the primary cohort included 22 eyes and the expanded cohort included 62 eyes. The mediation patterns remained remarkably consistent with the main analysis. In the primary cohort, IT pVD continued to significantly mediate all three VF outcomes (MD, PSD, VFI), with nearly identical indirect effect estimates. In the expanded cohort, both IT and ST pVD remained significant mediators for PSD and VFI, with IT also mediating MD.

Given that axial elongation alters OCTA scan magnification and could induce global reductions in pVD measurements, we repeated mediation analyses using magnification-resistant relative pVD mediators (defined as sector pVD minus the mean of the other seven sectors). This approach removes variance attributable to uniform global shifts while preserving sector-specific signals. In the primary cohort, relative IT pVD showed attenuated but directionally consistent mediation effects for all three VF outcomes, with confidence intervals approaching statistical significance for the PSD (indirect effect = 0.565, 95% CI [−0.008, 1.131]) and showing marginal effects for MD (indirect effect = −0.518, 95% CI [−1.278, 0.077]) and VFI (indirect effect = −1.485, 95% CI [−3.222, 0.138]). In the expanded cohort, relative mediator effects were similarly directionally consistent but did not reach statistical significance.

Taken together, IT pVD emerged as the most consistent mediator of the relationship between AL and all three VF outcomes across both cohorts and sensitivity analyses, with ST pVD providing additional support in the expanded cohort.

## 4. Discussion

Glaucoma is a multifactorial optic neuropathy in which both biomechanical and vascular mechanisms contribute to disease progression. While prior studies have demonstrated associations between myopia, reduced pVD, and glaucomatous damage, the present pilot study attempts to formally quantify the extent to which pVD mediates the AL–VF damage relationship using mediation analysis. This approach moves beyond pairwise associations to estimate sector-specific indirect pathways, providing preliminary mechanistic insight into what has previously been described only in associative terms. Our study demonstrates that pVD significantly mediates the relationship between AL and VF damage in this patient cohort with POAG, supporting the hypothesis that axial elongation contributes to glaucomatous injury not only through mechanical strain but also via microvascular compromise.

We emphasize that our primary findings derive from the cohort with measured AL (*n* = 26 after quality exclusions), with the expanded cohort analysis (*n* = 71) serving to explore pattern consistency rather than provide true external validation. We identified significant indirect effects of AL on all three VF outcomes (MD, PSD, and VFI) mediated by IT pVD (Table 3). This sector demonstrated the strongest mediation, suggesting it plays a central role in the biomechanical–vascular pathway linking axial elongation to glaucomatous damage. ST pVD also emerged as a significant mediator in our expanded cohort (Table 4), reinforcing the importance of arcuate sector perfusion in POAG. We confirmed that multicollinearity did not meaningfully influence our mediation models, as all VIF values were well below accepted thresholds. This supports the stability of our regression estimates and suggests that the observed associations between AL, sectoral pVD, and VF outcomes are unlikely to be artifacts of correlated predictors.

Our findings support the biological plausibility that AL influences glaucoma outcomes via microvascular compromise, particularly in the ONH region. Elevated IOP appears to exacerbate microvascular compromise in glaucomatous eyes, particularly when AL is longer. In one study, higher IOP predicted lower pVD in glaucoma (*p* = 0.009), with the effect magnified in eyes with AL ≥ 23.46 mm (*p* = 0.005) but absent in those with shorter AL (<23.46 mm, *p* = 0.45) and healthy controls (*p* = 0.26) [18].

The mediation effects observed in the IT sector align with prior studies demonstrating that IT pVD consistently shows the strongest correlation with glaucomatous damage. IT pVD has been shown to be a robust diagnostic and monitoring marker in glaucoma, with strong associations with RNFL thickness, achieving an area under the curve (AUC) of up to 0.88 in POAG and 0.86 in primary angle closure glaucoma, with sensitivities at 95% specificity reaching 70% and 67%, respectively [19]. It also correlates strongly with visual function, with R^2^ values up to 0.58 for IT pVD and superior VF loss (*p* < 0.001) [20]. In non-myopic eyes, reduced IT pVD in regions with RNFL defects is more pronounced in perimetric glaucoma compared to pre-perimetric stages, suggesting a link between microvasculature and functional loss in this region [21]. In early NTG, the IT sector demonstrates the strongest vascular-functional correlation, with VD showing consistent associations with VF loss across all disease stages, whereas RNFL correlates only in moderate-to-severe NTG, highlighting the significance of the IT sector and the role of VD in early disease detection [22]. In highly myopic POAG eyes with superior hemifield defects, IT pVD was significantly reduced compared to controls (mean 44.7% vs. 58.8%, *p* < 0.001), while still maintaining strong correlations with RNFL thickness (R = 0.749, *p* < 0.001) and VF loss (R = 0.587, *p* < 0.001) [23]. The ST sector also demonstrates strong correlations between VD and VF loss, further supporting the findings in our expanded cohort analysis [24,25].

Our findings align with the current literature, indicating that pVD is significantly associated with visual field sensitivity (VFS) in myopic glaucomatous eyes. In a retrospective cross-sectional study comparing 82 high myopes (75 with early glaucoma) to 122 non-high myopes (111 with early glaucoma), both global and sectoral pVD showed significant correlations with corresponding VFS across all 24-2 and 10-2 VF sectors (all *p* < 0.05) [26]. Furthermore, the pVD-VFS relationship appears to be stronger than the RNFL-VFS relationship in glaucomatous eyes with high myopia. A prospective cross-sectional analysis of 48 glaucomatous eyes with high myopia and 82 without high myopia revealed that global pVD-VFS associations were significantly stronger than RNFL-VFS associations in the high myopia group (*p* = 0.009), with no significant difference observed in the non-high myopia group (*p* = 0.343) [27].

Similarly, in a prospective cross-sectional study looking at 81 Japanese patients with open-angle glaucoma, the associations of MD and VFI with pVD were consistently stronger than those with RNFL thickness across all refractive error categories. Notably, while RNFL-VF correlations were only significant in hyperopic/emmetropic and moderate myopia groups, pVD showed consistently strong-to-very strong, statistically significant correlations with both MD and VFI in all refractive subgroups, with R-values ranging from 0.548 (*p* = 0.005) to 0.841 (*p* < 0.001). Additionally, PSD showed significant associations with pVD in mild (R = −0.572, *p* = 0.013), moderate (R = −0.545, *p* = 0.013), and high myopia (R = −0.538, *p* = 0.018), further reinforcing the relevance of pVD as a functional correlate and supporting our use of MD, VFI, and PSD in our mediation analysis [28].

Interestingly, in several models, the direct effect of AL on VF outcomes remained paradoxically protective after accounting for VD, as evidenced by the opposite signs of the indirect effect and the direct effect (c′) (Table 3 and Table 4). This phenomenon is most likely a statistical artifact known as suppression or inconsistent mediation, which can occur in mediation models with small sample sizes and strong correlations between predictor and mediator variables. Given our small sample size, these paradoxical direct effects should be interpreted with caution as they likely represent statistical phenomena rather than true biological protective mechanisms. While some prior research has reported slower VF progression in myopic glaucomatous eyes, the evidence remains inconsistent and preliminary [29,30]. Possible mechanisms have been proposed, including IOP fluctuation due to stiffer sclera, biomechanical remodeling of the lamina cribrosa, and sectoral RNFL preservation [31,32,33]. However, our primary inference should focus on the robust indirect effects (vascular mediation) rather than these unreliable direct effects, which may not replicate in adequately powered studies

The magnification sensitivity analysis revealed that sector-specific mediation effects, particularly in the IT region, persist even after removing global pVD shifts attributable to AL-dependent magnification. While these relative mediator effects were attenuated compared to absolute values (reflecting shared variance removal and reduced power), their directional consistency and near-significant effects in the primary cohort suggest that the observed mediation is not purely a magnification artifact. Rather, the findings indicate that both mechanisms contribute: magnification induces global reductions across all sectors, while glaucomatous damage produces additional focal deficits in vulnerable regions such as the IT arcade. This interpretation aligns with the known susceptibility of inferior sectors to glaucomatous damage and supports a biomechanical-vascular model wherein axial elongation acts through both geometric (magnification) and pathophysiological (mechanical strain on vessels) pathways.

Our pilot findings provide insight into the vascular mechanisms linking AL to glaucomatous damage, but several limitations must be acknowledged. Non-glaucomatous controls were excluded from mediation analyses by design: the outcome variable (glaucomatous VF damage) is undefined in healthy eyes, making their inclusion in the mediation framework inappropriate rather than a methodological oversight. This does restrict our ability to isolate glaucoma-specific vascular effects from those attributable to axial elongation alone, and future studies incorporating a parallel analysis in non-glaucomatous high myopes would strengthen causal inference. Importantly, the mediation observed in our models was partial, not complete. This implies that while pVD explains a significant portion of the relationship between AL and VF outcomes in glaucoma, other pathways likely contribute as well. The relatively small sample size of the primary cohort (*n* = 26) is a limitation that warrants careful interpretation. While smaller than ideal for mediation analysis, our variance inflation factor (VIF) analysis demonstrated no concerning multicollinearity (all VIF < 2.0), indicating stable regression estimates. Bias-corrected bootstrap confidence intervals (5000 resamples) were used throughout, as this approach is specifically recommended for mediation analysis in small samples due to its robustness to distributional assumptions. We reduced the number of covariates from our initial analysis to three (age, IOP, scan quality) to optimize the sample-to-parameter ratio. The expanded cohort (*n* = 71) demonstrated consistent mediation patterns, and sensitivity analyses confirmed robustness to quality thresholds. Nevertheless, these results should be considered hypothesis-generating, and confirmation in larger prospective cohorts with measured AL is warranted. Another significant limitation is the use of estimated AL for 45 of the 71 eyes (63%) in the expanded cohort. While our AL estimation model demonstrated good statistical performance (R^2^ = 0.626, *p* < 0.001), the 95% prediction interval of ±1.49 mm indicates substantial measurement uncertainty. Measurement error in the independent variable (AL) typically attenuates observed associations and reduces statistical power, which may explain some differences in mediation patterns between the primary and expanded cohorts. For this reason, we emphasize findings from the primary cohort with measured AL (*n* = 26) as our main results, with the expanded cohort serving to assess pattern consistency in an exploratory manner. True validation of these findings requires an independent cohort with directly measured AL. Additionally, AL was estimated using SE and age, without keratometry data. While this approach has been shown to yield reasonable estimates, studies incorporating keratometry demonstrate improved accuracy in AL prediction [34].

Separately, we did not apply post hoc magnification corrections to pVD measurements because such corrections require either raw image data or validated algorithms specific to the device’s pVD computation, neither of which was available in our dataset. pVD is an area fraction derived through complex image processing pipelines (segmentation, projection removal, thresholding), and these algorithms do not necessarily scale linearly with geometric corrections. Additionally, applying AL-derived magnification factors to correct the mediator would create statistical confounding, as the correction factor would be perfectly collinear with the exposure variable (AL). Instead, we employed a magnification-resistant relative mediator approach that isolates sector-specific signals from global shifts. While this analysis showed attenuated but directionally consistent effects, we acknowledge that magnification likely contributes to the observed associations. Future studies using OCTA devices with built-in magnification correction or access to raw scan geometry would allow more definitive separation of magnification from disease effects. We also employed a scan quality threshold of ≥4 as our primary inclusion criterion to maximize sample size while controlling for scan quality as a covariate in all models. Although sensitivity analysis with a stricter threshold (scan quality ≥ 6) yielded consistent results, inclusion of lower-quality scans may have introduced measurement noise that could attenuate observed relationships.

Diurnal variations in both IOP and OCTA measurements or other systemic or chronic disease processes that may be correlated with POAG represent other potential confounding factors of our analysis. Notably, we did not stratify analyses by anti-glaucoma medication class. Certain IOP-lowering agents, including prostaglandin analogs and beta-blockers, may modulate ocular blood flow and could represent an unmeasured confounder in the relationship between AL, pVD, and VF outcomes; future studies with sufficient power should account for medication class in their analyses. To optimize the sample-to-parameter ratio, mediation models were adjusted for age, IOP, and scan quality only. Including additional demographic covariates such as sex, race/ethnicity, or systemic comorbidities would have over-parameterized the models and risked multicollinearity; larger future studies should incorporate these variables. The mean AL in the primary cohort (24.62 ± 1.41 mm) was near the population average and did not predominantly consist of high myopes (typically defined as AL ≥26 mm). The study examines AL as a continuous predictor within a POAG population, and findings may not generalize specifically to high myopic glaucoma. Future work in cohorts enriched for high myopia is warranted. Additionally, distinguishing glaucomatous from myopia-related VF damage in eyes with longer AL remains an inherent limitation. The cross-sectional nature of the study precludes causal inference.

In summary, these results underscore the potential importance of vascular compromise in the pathophysiology of glaucomatous damage associated with longer AL. The data suggests that axial elongation may influence POAG through both biomechanical and microvascular pathways. While these pilot results are suggestive, significantly larger sample sizes with longitudinal observations that include non-glaucomatous controls are needed to validate these findings and clarify the temporal dynamics of vascular change and glaucomatous progression associated with longer AL.

## Figures and Tables

**Table 1:** Demographics, clinical data, and imaging data of primary and expanded cohorts. Scan quality score ranges from 0 (poorest) to 10 (best quality). Abbreviations: IOP, intraocular pressure; MAP, mean arterial pressure; CCT, central corneal thickness; OCT-A, optical coherence tomography angiography; MD, mean deviation; PSD, pattern standard deviation; VFI, visual field index.

	Primary Cohort (*n* = 26)	Expanded Cohort (*n* = 71)	*p*-Value
Demographics:			
Age (years)	72.42 ± 7.41	69.10 ± 8.88	0.0952
Sex:			0.5655
Female	13 (50%)	42 (59.2%)	
Male	13 (50%)	29 (40.8%)	
Race:			0.1709
White	15 (57.7%)	30 (42.3%)	
Black	6 (23.1%)	19 (26.8%)	
Asian	0 (0.0%)	6 (8.5%)	
Hispanic	0 (0.0%)	7 (9.9%)	
Other	5 (19.2%)	9 (12.7%)	
Laterality:			0.6360
Right	10 (38.5%)	33 (46.5%)	
Left	16 (61.5%)	38 (46.5%)	
IOP (mmHg)	15.19 ± 5.22	15.77 ± 4.57	0.5983
MAP (mmHg)	92.73 ± 8.76	94.27 ± 10.17	0.5006
CCT (µm)	540.42 ± 35.76	544.99 ± 36.90	0.5917
Axial length (measured and/or estimated, mm)	24.62 ± 1.41	24.55 ± 1.15	0.8250
OCT-A:			
Vessel density—SN	40.35 ± 7.17	39.87 ± 8.15	0.7965
Vessel density—NS	39.38 ± 8.38	40.90 ± 7.12	0.3834
Vessel density—NI	36.85 ± 7.66	38.82 ± 7.59	0.2662
Vessel density—IN	40.96 ± 8.53	40.03 ± 9.03	0.6516
Vessel density—IT	43.27 ± 11.89	44.93 ± 11.56	0.5397
Vessel density—TI	48.23 ± 6.49	46.65 ± 6.83	0.3136
Vessel density—TS	52.19 ± 7.00	51.24 ± 7.19	0.5659
Vessel density—ST	45.69 ± 10.60	46.15 ± 10.25	0.8473
Scan quality	7.08 ± 1.38	7.23 ± 1.34	0.6375
Visual Field:			
MD (dB)	−3.87 ± 3.77	−3.18 ± 4.07	0.4603
PSD (dB)	4.00 ± 2.97	3.68 ± 2.42	0.5984
VFI (%)	92.38 ± 8.65	92.65 ± 10.41	0.9095

**Table 2:** Demographics and clinical data of AL estimation model cohort. Abbreviations: OLS, ordinary least squares; IOP, intraocular pressure; MAP, mean arterial pressure; CCT, central corneal thickness.

	OLS AL Estimation Cohort (*n* = 27)
Demographics:	
Age (years)	70.41 ± 9.45
Sex:	
Female	12 (44.4%)
Male	15 (55.6%)
Race:	
White	11 (40.7%)
Black	8 (29.6%)
Asian	1 (3.7%)
Hispanic	2 (7.4%)
Other	5 (18.5%)
Laterality:	
Right	11 (40.7%)
Left	16 (59.3%)
Glaucoma	23 (85.2%)
IOP (mmHg)	14.74 ± 3.81
MAP (mmHg)	92.81 ± 9.52
CCT (µm)	539.48 ± 40.44
Axial length (mm)	24.5 ± 1.27
Spherical equivalent (D)	−0.81 ± 2.89

**Table 3:** Mediation analysis of primary cohort. Models adjusted for age, IOP, and scan quality. * indicates significant indirect effect (95% CI excludes zero).

	Mediator	a	b	c	c′	Indirect Effect	CI [2.5%, 97.5%]
Outcome = MD (*n* = 26)							
	Superior Nasal	−1.50654	0.22609	−0.57212	−0.23149	−0.34062	[−1.12895, 0.13821]
	Nasal Superior	−1.20630	0.30604	−0.57212	−0.20294	−0.36917	[−1.24062, 0.31907]
	Nasal Inferior	−3.18395	0.39175	−0.57212	0.67520	−1.24731	[−2.36297, −0.17043] *
	Inferior Nasal	−1.66335	0.24871	−0.57212	−0.15843	−0.41369	[−1.12665, 0.07816]
	Inferior Temporal	−4.35788	0.23901	−0.57212	0.46947	−1.04159	[−1.93845, −0.19832] *
	Temporal Inferior	−1.40205	0.21139	−0.57212	−0.27574	−0.29638	[−0.93316, 0.14619]
	Temporal Superior	−1.13540	0.28037	−0.57212	−0.25379	−0.31833	[−1.23339, 0.23056]
	Superior Temporal	−2.89009	0.22138	−0.57212	0.06768	−0.63980	[−1.44086, 0.08210]
Outcome = PSD (*n* = 26)							
	Superior Nasal	−1.50654	−0.11610	0.37277	0.19786	0.17491	[−0.14802, 0.62945]
	Nasal Superior	−1.20630	−0.17165	0.37277	0.16570	0.20707	[−0.19608, 0.74946]
	Nasal Inferior	−3.18395	−0.17551	0.37277	−0.18606	0.55883	[−0.21477, 1.50821]
	Inferior Nasal	−1.66335	−0.07425	0.37277	0.24926	0.12350	[−0.19396, 0.62518]
	Inferior Temporal	−4.35788	−0.19274	0.37277	−0.46719	0.83996	[0.14894, 1.58039] *
	Temporal Inferior	−1.40205	−0.20962	0.37277	0.07886	0.29390	[−0.15299, 0.83415]
	Temporal Superior	−1.13540	−0.15770	0.37277	0.19372	0.17905	[−0.12957, 0.64466]
	Superior Temporal	−2.89009	−0.17053	0.37277	−0.12008	0.49284	[−0.04058, 1.14855]
Outcome = VFI (*n* = 26)							
	Superior Nasal	−1.50654	0.53198	−1.68260	−0.88115	−0.80145	[−2.45594, 0.33306]
	Nasal Superior	−1.20630	0.60701	−1.68260	−0.95037	−0.73223	[−2.52383, 0.63589]
	Nasal Inferior	−3.18395	0.71018	−1.68260	0.57859	−2.26119	[−4.97368, 0.14552]
	Inferior Nasal	−1.66335	0.51918	−1.68260	−0.81903	−0.86358	[−2.56591, 0.13804]
	Inferior Temporal	−4.35788	0.60690	−1.68260	0.96219	−2.64480	[−4.66051, −0.59060] *
	Temporal Inferior	−1.40205	0.66473	−1.68260	−0.75062	−0.93199	[−2.51379, 0.40406]
	Temporal Superior	−1.13540	0.67179	−1.68260	−0.91985	−0.76275	[−2.63007, 0.48379]
	Superior Temporal	−2.89009	0.56786	−1.68260	−0.04143	−1.64118	[−3.56692, 0.18497]

**Table 4:** Mediation analysis of expanded cohort. Models adjusted for age, IOP, and scan quality. * indicates significant indirect effect (95% CI excludes zero).

	Mediator	a	b	c	c′	Indirect Effect	CI [2.5%, 97.5%]
Outcome = MD (*n* = 71)							
	Superior Nasal	−2.19500	0.15391	−0.25981	0.07804	−0.33784	[−0.86841, 0.08159]
	Nasal Superior	−0.98899	0.26801	−0.25981	0.00525	−0.26506	[−0.74469, 0.11632]
	Nasal Inferior	−2.14421	0.19659	−0.25981	0.16173	−0.42153	[−1.02564, −0.03166] *
	Inferior Nasal	−1.09992	0.14990	−0.25981	−0.09493	−0.16488	[−0.61591, 0.09851]
	Inferior Temporal	−2.33159	0.19311	−0.25981	0.19046	−0.45026	[−1.12660, −0.00717] *
	Temporal Inferior	−0.82508	0.23139	−0.25981	−0.06889	−0.19091	[−0.60878, 0.16404]
	Temporal Superior	−0.51333	0.28351	−0.25981	−0.11427	−0.14553	[−0.61849, 0.22208]
	Superior Temporal	−2.05410	0.20745	−0.25981	0.16631	−0.42611	[−1.04753, −0.02214] *
Outcome = PSD (*n* = 71)							
	Superior Nasal	−2.19500	−0.08527	0.20310	0.01594	0.18716	[−0.03949, 0.46858]
	Nasal Superior	−0.98899	−0.12908	0.20310	0.07544	0.12766	[−0.05859, 0.36302]
	Nasal Inferior	−2.14421	−0.08613	0.20310	0.01842	0.18468	[−0.02736, 0.50988]
	Inferior Nasal	−1.09992	−0.04888	0.20310	0.14934	0.05376	[−0.05376, 0.25162]
	Inferior Temporal	−2.33159	−0.12425	0.20310	−0.08660	0.28970	[0.00751, 0.70323] *
	Temporal Inferior	−0.82508	−0.12567	0.20310	0.09941	0.10368	[−0.08264, 0.34606]
	Temporal Superior	−0.51333	−0.13951	0.20310	0.13148	0.07162	[−0.10602, 0.29282]
	Superior Temporal	−2.05410	−0.13353	0.20310	−0.07118	0.27428	[0.00868, 0.70828] *
Outcome = VFI (*n* = 71)							
	Superior Nasal	−2.19500	0.54894	−0.73386	0.47106	−1.20491	[−2.95404, 0.03964]
	Nasal Superior	−0.98899	0.67616	−0.73386	−0.06514	−0.66871	[−2.04752, 0.30033]
	Nasal Inferior	−2.14421	0.48903	−0.73386	0.31472	−1.04857	[−2.73474, 0.00514]
	Inferior Nasal	−1.09992	0.45922	−0.73386	−0.22875	−0.50511	[−1.79080, 0.33807]
	Inferior Temporal	−2.33159	0.54315	−0.73386	0.53254	−1.2664	[−3.10045, −0.02719] *
	Temporal Inferior	−0.82508	0.67793	−0.73386	−0.17451	−0.55935	[−1.79609, 0.45655]
	Temporal Superior	−0.51333	0.80186	−0.73386	−0.32224	−0.41162	[−1.89547, 0.60005]
	Superior Temporal	−2.05410	0.59442	−0.73386	0.48714	−1.22100	[−2.99679, −0.05958] *

## Data Availability

The data presented in the study are included in the article; further inquiries can be directed to the corresponding author.

## Abbreviations

The following abbreviations are used in this manuscript:

AL	Axial length
CCT	Central corneal thickness
IOP	Intraocular pressure
MAP	Mean arterial pressure
ONH	Optic nerve head
RGC	Retinal ganglion cell
RNFL	Retinal nerve fiber layer
SE	Spherical equivalent
NTG	Normal tension glaucoma
POAG	Primary open angle glaucoma
OCT	Optical coherence tomography
OCTA	Optical coherence tomography angiography
SD-OCT	Spectral domain—optical coherence tomography
pVD	Peripapillary vessel density
VD	Vessel density
IN	Inferior-nasal
IT	Inferior-temporal
NI	Nasal-inferior
NS	Nasal-superior
SN	Superior-nasal
ST	Superior-temporal
TI	Temporal-inferior
TS	Temporal-superior
VF	Visual field
MD	Mean deviation
PSD	Pattern standard deviation
VFI	Visual field index
VFS	Visual field sensitivity
AUC	Area under the curve
BP	Blood pressure
CI	Confidence interval
OLS	Ordinary
VIF	Variance inflation factor
